# Characteristics of the MicroRNA Expression Profile of Exosomes Released by Vero Cells Infected with Porcine Epidemic Diarrhea Virus

**DOI:** 10.3390/v14040806

**Published:** 2022-04-13

**Authors:** Lei Yin, Xuehuai Shen, Dongdong Yin, Jieru Wang, Ruihong Zhao, Yin Dai, Xiaocheng Pan

**Affiliations:** 1 Livestock and Poultry Epidemic Diseases Research Center of Anhui Province, Institute of Animal Husbandry and Veterinary Science, Anhui Academy of Agricultural Sciences, Hefei 230031, China; yinlei1989@yeah.net (L.Y.); xuehuaishen1986@163.com (X.S.); yindd160@163.com (D.Y.); wangjr0317@163.com (J.W.); zrhkdy@aliyun.com (R.Z.); daiyin2020@163.com (Y.D.); 2 Anhui Province Key Laboratory of Livestock and Poultry Product Safety Engineering, Hefei 230031, China

**Keywords:** porcine epidemic diarrhea virus, exosome, microRNAs, host–pathogen interactions

## Abstract

Exosomes are nanoscale vesicles actively secreted by a variety of cells. They contain regulated microRNA (miRNA), allowing them to function in intercellular communication. In the present study, the role of exosomal miRNAs in porcine epidemic diarrhea virus (PEDV) infection was investigated using exosomes isolated from Vero cells infected with PEDV. The results of transmission electron microscopy observation showed that the exosomes are spherical in shape, uniform in size, and negatively stained in the membrane. Nanoparticle tracking analysis showed that the average exosome particle size is 130.5 nm. The results of miRNA sequencing showed that, compared with the control group, a total of 115 miRNAs are abnormally expressed in the exosomes of infected cells. Of these, 80 miRNAs are significantly upregulated and 35 miRNAs are significantly downregulated. Functional annotation analysis showed that the differentially expressed miRNAs are associated with PEDV infection through interaction with the cAMP, Hippo, TGF-beta, HIF-1, FoxO, MAPK, and Ras signaling pathways. Thus, our findings provide important information about the effects of PEDV infection on exosomal miRNA expression and will aid the search for potential anti-PEDV drug candidates.

## 1. Introduction

A severe intestinal disease caused by porcine epidemic diarrhea virus (PEDV), porcine epidemic diarrhea, is highly contagious. The pig industry has suffered enormous losses since it reappeared in 2010 [1]. PEDV is a single-stranded, envelope positive RNA virus of the coronavirus family (*Coronaviridae*). Infection with PEDV in suckling piglets can lead to severe enteritis, vomiting, and watery diarrhea, and its mortality rate for piglets under 1 week old is as high as 90% [2,3]. However, despite its severity, the biological mechanisms of PEDV infection, especially the interactions between host and pathogen, are largely unknown. Therefore, there is an urgent need to understand the pathogenesis of PEDV and thus provide information vital for the development of suitable antiviral drugs.

Exosomes are a kind of microvesicle structure with diameters of 30–100 nm. They can be secreted by almost all types of cells and tissues [4,5]. Exocrine vesicles can carry a variety of proteins, functional RNAs, and lipids as well as other bioactive substances and can transfer these bioactive substances from origin cells to target cells, thus affecting the regulation of biochemical components and signaling pathways in the target cells [6,7]. Accordingly, exosome research is receiving widespread attention in the field of virus infection.

It has been found that the exosomes secreted by host cells after virus infection carry active virus or host-cell components and can regulate the immune response of target cells or cause target cell infection [8,9]. For example, the exosomes of the hepatitis A virus are endowed with an unenveloped virus membrane, allowing the virus to escape immune recognition by the host [10]. Furthermore, exosomes from non-parenchymal liver cells can transfer the antiviral activity induced by interferons to hepatocytes replicated by the hepatitis B virus [11]. Thus, exosomes are involved in the life cycles of many viruses.

MicroRNAs (miRNAs) are small, noncoding regulatory RNA molecules with lengths of ~22 nucleotides; they are the most abundant RNA in exosomes [12,13]. In interactions between a host and a virus, exosomes can specifically select miRNAs in packaging cells to directly target virus genomic RNA and inhibit virus replication, thus playing an important regulatory role [14,15]. For example, exosomes isolated from HeLa cells infected with Newcastle disease virus promote its spread by carrying miRNA into adjacent cells [16]. Furthermore, exosomes released by prion-infected nerve cells show significantly increased levels of miR-128a, miR-21, miR-222, miR-29b, miR-342-3p, and miR-424 compared to those of uninfected exosomes [17]; in exosomes infected with HIV, the expressions of miR-29a, miR-150, miR-518, and miR-875 are upregulated 16- to 44-fold [18]. These differentially expressed miRNAs have been shown to be involved in virus replication and reproduction [19,20]. However, the role of exocrine miRNAs in the pathogenesis of PEDV infection is not clear.

In this study, the exosomes released by Vero cells infected with PEDV were identified and the differential expression of miRNAs therein was investigated. This work lays a foundation for further study of the role of miRNAs in the pathogenesis of PEDV.

## 2. Materials and Methods

### 2.1. Cells and Virus

A culture of Vero cells (CVCCCL28, purchased from the China Institute of Veterinary Drug Control (Beijing, China)) was performed in Dulbecco’s Modified Eagle’s Medium (Gibco BRL, Grand Island, NY, USA) supplemented with 10% fetal bovine serum (HyClone, Logan, UT, USA) and 1% penicillin-streptomycin. The Jiangsu Academy of Agricultural Sciences provided the classical PEDV CV777 strain (GenBank: KT323979.1) for this study.

### 2.2. Exosome Isolation

Vero cells were infected with PEDV at a multiplicity of infection (MOI) of 1 and cultured for 24 h. Exosomes were isolated and purified from a PEDV-infected Vero cell culture supernatant 24 h post infection according to the following protocol: Exosomes were isolated from the supernatants of the cells by differential centrifugation according to Thery et al. [21]. Briefly, in the first step, the supernatant of the culture medium was transferred to a centrifuge tube and the large vesicles were removed by centrifugation at 4 °C for 45 min. In the second step, the supernatant was filtered through a 10,000-μm membrane; the filtrate was transferred to a new centrifuge tube and subjected to centrifugation at 100,000× *g* and 4 °C for 70 min. Then, the supernatant was re-suspended in 10 mL of precooled 1× PBS. Finally, the supernatant was removed by ultracentrifugation at 100,000× *g* for 70 min at 4 °C, resuspended in 150 μL of precooled 1× PBS, and stored at −80 °C.

### 2.3. Transmission Electron Microscopy (TEM)

In order to observe the morphology of exosomes, a 0.2% paraformaldehyde suspension was mixed with an exosome suspension, which was then applied to a formvar-coated copper grid. Staining with uranyl acetate 1% in aqueous water for 2 min was followed by filtering the liquid off and examining the sample under an electron microscope (FEI, Hillsboro, OR, USA).

### 2.4. Nanoparticle Tracking Analysis (NTA)

The concentration and size distribution profile of the exosomes were measured using a NanoSight NS300 system (Malvern Instruments Ltd., Malvern, UK) and the data were analyzed with NTA 3.1 Dev Build 3.1.54 software. We resuspended exosome preparations in sterile PBS and then vortex emulsified them.

### 2.5. MiRNA Microarray Assay and Bioinformatics Analysis of Target Genes

The Oe Biotech Corporation performed an miRNA profiling study of the exosomes of PEDV-infected Vero cells (Shanghai, China, http://www.oebiotech.com, accessed on 26 January 2022) [22].Briefly, in order to normalize the raw data, Genespring software was used to isolate miRNAs that were differentially expressed after RNA was extracted and labeled with an Agilent-070154 Rat miRNA Microarray V21.0 8 × 15K (Agilent, Santa Clara, CA, USA) [23,24]. DEmiRNAs targeting up- and downregulated genes were chosen using two intersections of two databases (Targetscan and microRNAorg) that showed a fold change of ≥1.5 and a *p* ≤ 0.05 [25]. Gene ontology (GO) and the Kyoto Encyclopedia of Genes and Genomes (KEGG) were used to analyze functional and pathway enrichment in putative genes. A *p* ≤ 0.05 was defined as the threshold of significance for GO and KEGG analyses, respectively [26].

### 2.6. Analysis of the miRNAs by qRT-PCR

The expression levels of miRNAs were identified by sequencing and qRT-PCR assay. MiRcute miRNA qPCR SYBR Green Detection Kit (Vazyme, Nanjing, China) was utilized with an ABI Step One thermocycler (Applied Biosystems, Foster City, CA, USA) for qRT-PCR. The miRNA-specific forward primers used in this study are shown in Table 1. The U6 snRNA was used as an internal standard. Three independent biological replicates were used for each gene. The relative expression level of each miRNA was calculated by the 2^−ΔΔct^ method [27].

### 2.7. Statistical Analysis

All statistical analyses were performed with SPSS 21.0 statistical software. Data are presented as means ± SD. In this study, we compared groups using one-way analysis of variance (ANOVA), followed by a post hoc comparison using the least significant difference (LSD). A *p* < 0.05 was considered statistically significant.

## 3. Results

### 3.1. Characterization of Exosomes

**Figure 1:**
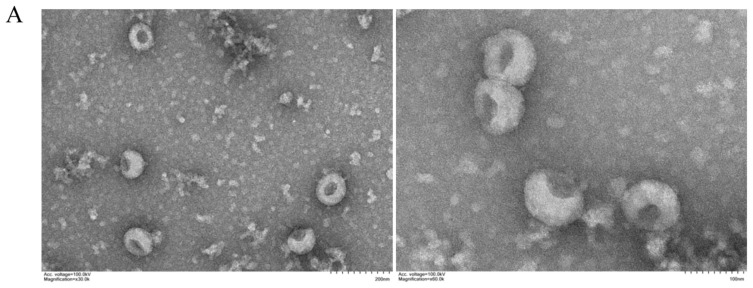

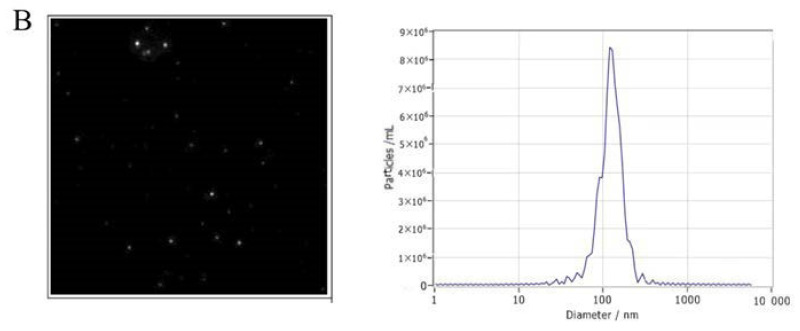
Characterization of Vero cell-derived exosomes. Exosomes were isolated and purified from PEDV-uninfected and -infected Vero cell culture. (**A**) Morphology of exosomes observed by TEM. Scale bars, 100 nm and 200 nm. (**B**) Particle size and quantification analysis of exosomes by NTA.

In order to analyze the exosomes, we used TEM and NTA. The TEM revealed round vesicle structures ranging in size from 30 to 200 nm (Figure 1A). According to NTA measurements, the size distribution peak was found at a 130.5-nm diameter (Figure 1B), which is consistent with the previously reported characteristics of exosomes. All these data indicate the successful isolation of exosomes.

### 3.2. Analysis of Small RNA Sequencing Library Data

**Figure 2:**
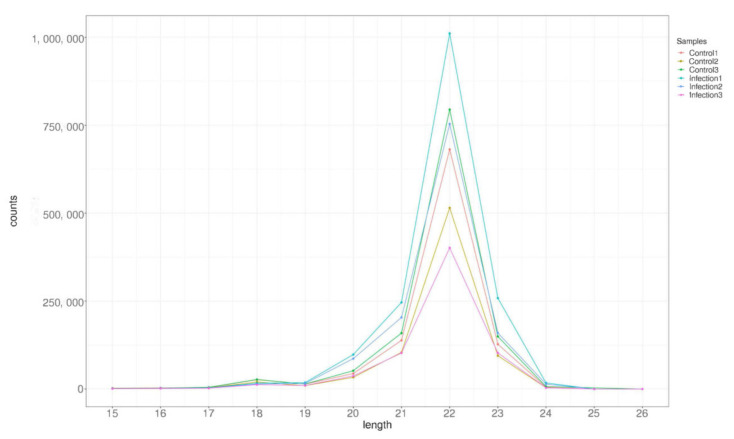
Clean read length distribution on each sequence. The x-axis represents the read length. The y-axis represents the percentage of each read length.

Six microRNA libraries were constructed from PEDV-infected and control Vero cells and sequenced to reveal the effects of PEDV infection on exosomal miRNAs. A total of 30,572,744, 26,165,923, and 27,571,400 raw reads were obtained from infected (Infections 1,2, and 3) cells, shown in Table 2, while 27,070,230, 21,158,763, and 26,987,232 were obtained from uninfected (Controls 1,2, and 3) cells. After removing low-quality tags, adapter sequences, and short reads smaller than 15 nt, 24,269,195, 21,385,578, and 22,875,953 (infected) and 20,920,004, 16,414,523, and 20,535,191 (uninfected) clean reads were identified. Further, the data were divided into the following categories: miRNA, rRNA, snRNA, tRNA, Cis-region, repeat, other Rfam-RNA, and unannotated (Table 2). The length distribution of the miRNA is presented in Figure 2. From all the libraries, most miRNAs had a length of 22 nt.

### 3.3. Identification of Known MiRNAs in Exosomes

**Figure 3:**
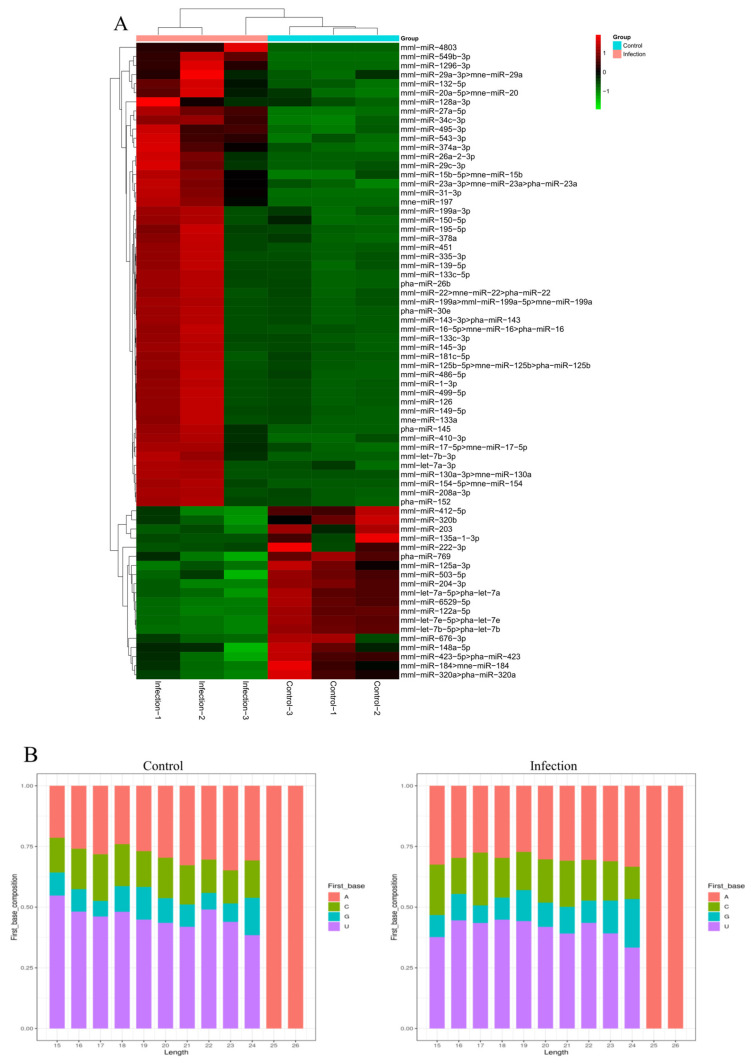
Differential expression levels of known miRNAs. (**A**) Hierarchical clustering analysis of known miRNAs in the PEDV-infected and control groups using the R program. Euclidean methods and complete linkage were used for this analysis. (**B**) Infected and uninfected cells have different sizes and base biases of miRNA at the first position. MiRNA lengths are given on the x-axis between 15and 26 nucleotides. MiRNA base bias is represented as a percentage at the first position of the y-axis.

Identification of known miRNAs that are altered when Vero cells are infected with PEDV, an miRNA count, and a base bias at the first position were obtained by mapping the small RNA sequences to the mature miRNAs and their precursors in the miRBase 20 database. An estimated 2074, 2003, and 1752 unique sequences (1,673,115, 1,253,338, and 675,418 reads) were annotated as miRNA candidates in the infected library and 1386, 1208, and 1352 unique sequences (1,047,301, 788,983, and 1,212,878 reads) in the uninfected library (Table 2). PEDV-infected Vero cells were found to contain 441, 415, and 396 known miRNA genes while control-uninfected Vero cells contained 352, 326, and 346 known miRNA genes. A heat map of the miRNA expression patterns in the two groups can be seen in Figure 3A. The two groups were cut off at a *p* < 0.05 and |log2 (PEDV-infected/control-uninfected in expression)|>1. There were 70 known DEmiRNAs in the two groups, out of which 51 were upregulated and 19 were downregulated. Additionally, 22 nt appeared to be the dominant length for miRNAs, and the first nucleotide bias in the identified miRNAs clearly favored ′U′ at the 5′-end (Figure 3B).

### 3.4. Identification of Novel MiRNAs in Exosomes

**Figure 4:**
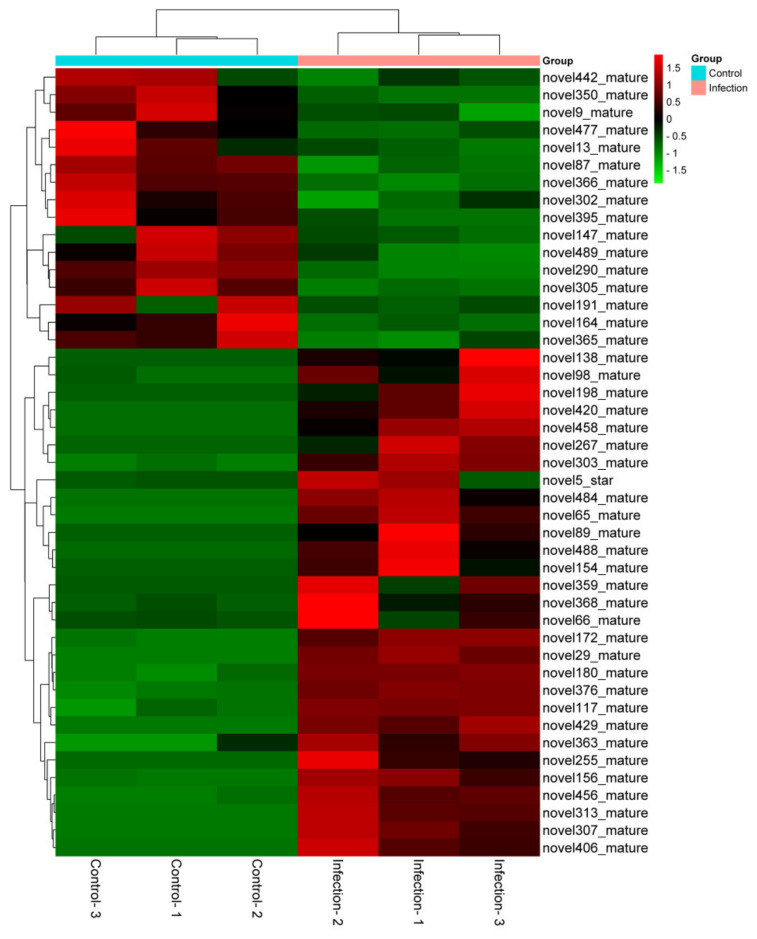
Using the R program, hierarchical clustering was used to determine novel miRNAs among PEDV-infected and control groups. Euclidean methods and complete linkage were used for this analysis. Upregulated and downregulated miRNAs are marked in red and green, respectively.

The PEDV-infected and uninfected groups contained 9,493,236, 8,307,505, and 8,358,603 and 9,414,281, 7,302,434, and 9,459,270 unannotated sRNAs, respectively; based on these sRNAs, new candidates for miRNAs were predicted. According to Table 2, miReap software predicted 310, 306, and 290 and 210, 181, and 208 novel miRNAs in the PEDV-infected and uninfected Vero cell libraries, respectively. As a result of the differential expression analysis, 45 novel miRNAs were identified in the two groups using the cut-off values reported previously, where 29 miRNAs were upregulated and 16 were downregulated (*p* < 0.05). The heat map in Figure 4 illustrates the differences in miRNA expression between the two groups.

### 3.5. Target Gene Prediction and Pathway Enrichment Analysis of DEmiRNAs

**Figure 5:**
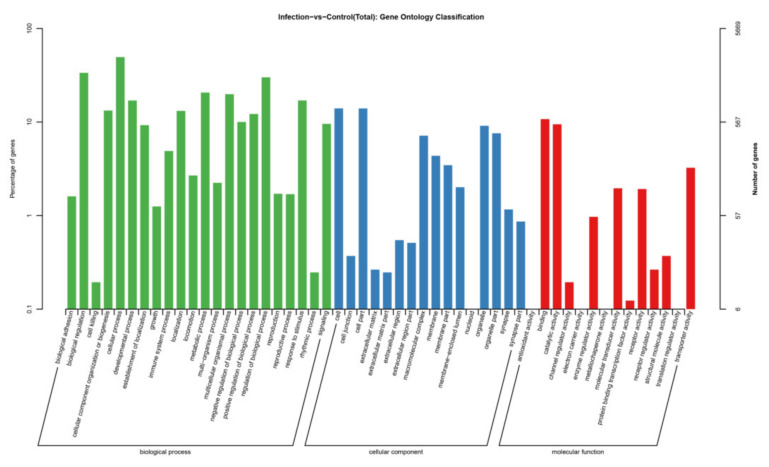
GO analysis of the target genes of the dysregulated miRNAs.

**Figure 6:**
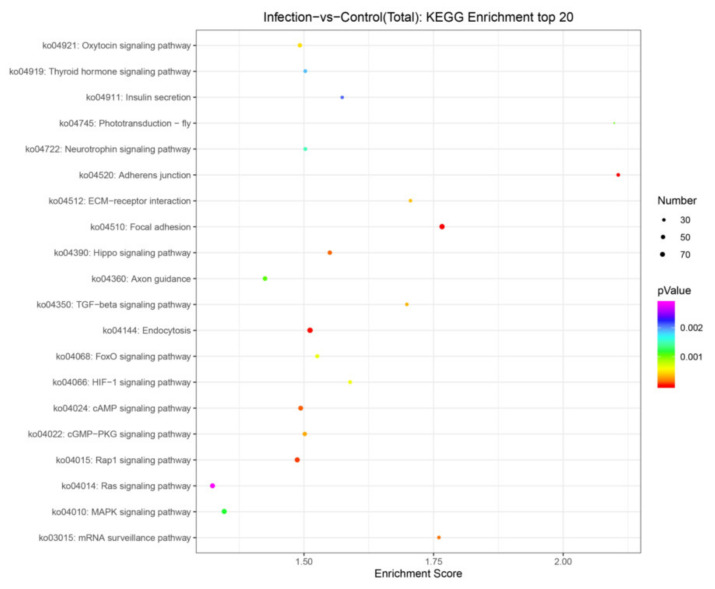
Top 20 KEGG pathways of the target genes of the differentially expressed miRNAs.

We compared the potential mRNA targets of two independent miRNA prediction algorithms, miRanda and RNAhybrid, to determine their biological functions. A total of 5282 genes for the 115 miRNAs was predicted as potential miRNA targets. GO analysis of the predicted target genes revealed that they are involved in the biological process, cellular component, and molecular function (Figure 5). KEGG orthology-based annotation system (KOBAS) analysis was carried out to analyze miRNA roles in regulatory networks. It was found that many of the abundant KEGG terms relate to biological processes including adherens junction (ko04520), focal adhesion (ko04510), endocytosis (ko04144), the MAPK signaling pathway (ko04010), the Hippo signaling pathway (ko04390), the mRNA surveillance pathway (ko03015), the TGF-beta signaling pathway (ko04350), ECM–receptor interaction (ko04512), the HIF-1 signaling pathway (ko04066), and the FoxO signaling pathway (ko04068) (Figure 6).

### 3.6. Validation of MiRNAs by qRT-PCR

**Figure 7:**
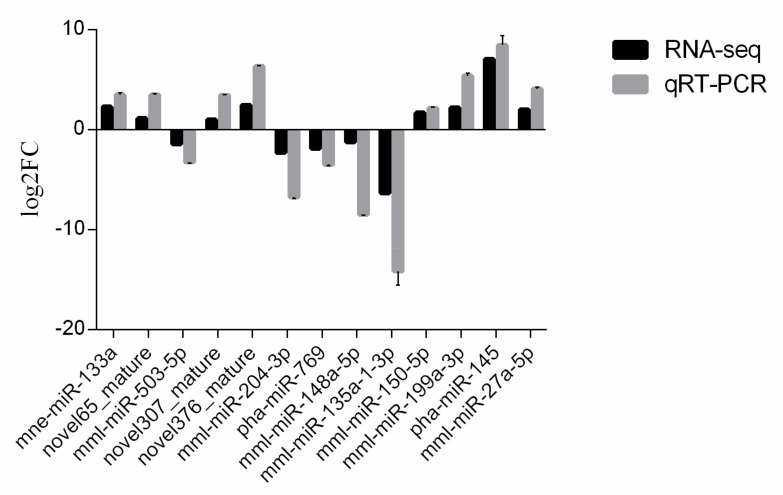
Validation of exosomal miRNAs’ expression by qRT-PCR.

An analysis of miRNAs differentially expressed was conducted using qRT-PCR assays based on the sequencing data. Three novel candidate miRNAs were selected for validation along with 10 known miRNAs. Compared with the sequencing data, the expression profiles were consistent. The downregulation of five miRNAs (mml-miR-503-5p, mml-miR-204-3p, pha-miR-769, mml-miR-148a-5p, and mml-miR-135a-1-3p) and the upregulation of eight miRNAs (mne-miR-133a, novel65_mature, novel307_mature, novel376_mature, mml-miR-150-5p, mml-miR-199a-3p, pha-miR-145,and mml-miR-27a-5p) in infected Vero cells compared with those in uninfected cells were confirmed (Figure 7).

## 4. Discussion

PEDV is a coronavirus that causes acute and highly contagious intestinal infectious diseases in piglets [28]. PEDV infection leads to dynamic changes of miRNA expression in the host cells and forms a complex interaction network with the virus [29,30]. In recent years, the use of high-throughput sequencing techniques to reveal the integration of miRNAs and mRNAs in viral infection has proven to be helpful in elucidating the regulatory mechanism of miRNA. However, it is not clear whether the miRNAs in exosomes affect PEDV replication by regulating host immune response and targeting viruses. Accordingly, we collected and observed the exosomes from PEDV-infected Vero cells. It is well known that Vero cells are the best host cells for PEDV isolation, passage, and experimental research in vitro [31]. Therefore, this study used Vero cells as the research object to explore the miRNA profiles of exosomes and how they are affected by PEDV infection.

Studying miRNAs in Vero cell exosomes after PEDV virus infection is an essential step to gaining insight into the role of miRNAs in intracellular communication and induction of antiviral responses. We obtained and successfully identified 70 known miRNAs and 45 novel miRNAs that are differentially expressed in PEDV-infected exosomes. These miRNAs may be involved in the interaction of Vero cells with PEDV. In the present study, most of the clean reading fragments in PEDV-infected and uninfected cells were 21 to 24 nt in length, with 22-nt RNA being the most abundant. These results are consistent with the typical size of miRNAs in Diller-derived products [32], indicating a high enrichment of miRNA sequences in the library.

An increasing number of studies have shown that exosomal miRNAs of host cells, through positive or negative regulation of host immunity, play a key role in virus transmission and immune evasion. In the present study, we found that the expression levels of mml-miR-148a-5p, mml-miR-423-5p, and mml-miR-135a-1-3p are significantly downregulated during PEDV infection while mml-miR-143-3p, mml-miR-150-5p, mml-miR-15b-5p, mml-miR-199a, pha-miR-145, mml-miR-23a, and mml-miR-27a expression levels are significantly upregulated during PEDV infection. It has been reported that DEF-cell-derived exosomal miR-148a-5p promotes duck Tembusu virus replication by negatively regulating TLR3 expression [33]. Furthermore, miR-143-3p, miR-150-5p, and miR-15b-5p show high expression levels in serum exosomes infected with Influenza A and B viruses [34], and in exosomes infected with Hepatitis C virus (HCV), the high expression of miR-199a and miR-145 promotes HCV RNA replication [35]. Human immunodeficiency virus (HIV)-infected macrophages secrete exosomes with high expression levels of miR-23a and miR-27a that disrupt the integrity of lung epithelial cells and mitochondrial biological functions [36]. In the exosomes secreted by the human diploid cell line Medical Research Council-5 (MRC-5), rabies virus infection upregulates microRNA (miR)-423-5p expression by abrogating the inhibition of cytokine signaling 3 (SOCS3) on type I interferon (IFN) signaling, resulting in feedback inhibition of RABV replication [37]. Furthermore, miR-135a family expression is downregulated to activate the p38 mitogen-activated protein kinase (MAPK)/p53 pathway, thereby contributing to apoptosis [38].

Recently, Han Zhao and colleagues identified that, after infecting Vero-E6 cells, PEDV downregulates expression of miRNA-328-3p and the resulting reduced inhibition of the target tight junction protein 3 (TJP-3/ZO-3) helps to enhance PEDV infection [39]. However, our results showed that transcript expression of miRNA-328-3p had no significant differential changes in exosomes released from PDEV-infected Vero cells. It was reported that miRNA expression is cell and specie specific, and the miRNAs are differentially expressed in exosomes released by different types of cells and cells in different physiological states, which also have varying effects on viral replication and its pathogenesis [40]. Therefore, our results suggest that these differentially expressed miRNAs may be involved in host–virus interactions in PEDV-infected Vero cells. However, it is not fully clear whether the miRNAs reported in this study are necessarily beneficial to understanding the participation of miRNA in exosomes from pigs. Therefore, further work is required.

The target genes of 115 miRNAs were predicted and the miRNAs were screened by qRT-PCR analysis. Based on the sRNA sequencing results, 13 miRNA expression profiles were consistent. In general, an miRNA has hundreds of predicted target genes, and a single target gene can be regulated by multiple miRNAs. In the present study, all the predicted mRNA transcripts were classified and annotated using GO and KEGG databases. GO analysis showed that the mRNA targets negatively associated with miRNAs are involved in biological regulation, immune system processes, responses to stimuli, and other cellular processes. Signaling pathway analyses conducted by KEGG revealed that the target genes are primarily involved in important cellular signaling pathways, including the cAMP signaling pathway, Hippo signaling pathway, TGF-beta signaling pathway, and the HIF-1 signaling pathway, indicating their important functions in the defense against PEDV infection. It was found that a host’s antiviral response depends on the control of various signaling pathways and that viruses evade cytosolic sensing by disrupting signaling pathways. For example, phosphodiesterase-induced cAMP degradation restricts hepatitis B virus infection [41]. The Hippo signaling pathway plays a key role in regulating viral replication [42]. The viral liver disease is accelerated by the transforming growth factor beta (TGF-β) by regulating viral progression and mediating inflammation-related responses [43]. Hypoxia inducible factor-1α (HIF-1α) is activated in host cells during viral infection and plays an important role at the site of inflammation by inducing the production of pro-inflammatory cytokines by immune cells [44]. Japanese encephalitis virus induces apoptosis by inhibiting the FoxO signaling pathway [45]. SV40 polyomavirus activates the Ras-MAPK signaling pathway for vacuolization, cell death, and virus release. Amyloid β (Aβ) deposition is a characteristic feature of human immunodeficiency virus-1 (HIV-1)-infected brains [46]. The Ras signaling pathway is involved in HIV-1-induced blood–brain barrier disruption, and Aβ deposition also plays an important role [47]. Therefore, targeting this pathway by specific miRNAs could be a promising therapeutic strategy to limit PEDV replication in target or neighboring cells.

## 5. Conclusions

In summary, we identified a number of dysregulated miRNAs in exosomes released from PEDV-infected Vero cells. The functions of these dysregulated miRNAs remain to be investigated in future studies, potentially helping us to elucidate the mechanisms of PEDV–host interactions.

## Figures and Tables

**Table 1:** Primers used to confirm miRNA expression with qRT-PCR.

MiRNA Name	MiRNA Sequence (5′-3′)	RT Primer Sequence (5′-3′)	Forward PCR Primer Sequence (5′-3′)
mne-miR-133a	TTGGTCCCCTTCAACCAGCTGT	GTCGTATCCAGTGCGTGTCGTGGAGTCGGCAATTGCACTGGATACGACACAGCT	CTCATTGGTCCCCTTCAACC
novel65_mature	GGTGGGGTCGGCGGGGGG	GTCGTATCCAGTGCGTGTCGTGGAGTCGGCAATTGCACTGGATACGACCCCCCC	TCATTATAGGTGGGGTCGGC
mml-miR-503-5p	TAGCAGCGGGAACAGTTCTGCAG	GTCGTATCCAGTGCGTGTCGTGGAGTCGGCAATTGCACTGGATACGACCTGCAG	ACTTAGCAGCGGGAACAGTT
novel307_mature	CGGCGGCGACGGTGGCGG	GTCGTATCCAGTGCGTGTCGTGGAGTCGGCAATTGCACTGGATACGACCCGCCA	TATATTTACGGCGGCGACGG
novel376_mature	CAGGGGTGGAGCCTGCGGA	GTCGTATCCAGTGCGTGTCGTGGAGTCGGCAATTGCACTGGATACGACTCCGCA	ATTACTTCAGGGGTGGAGCC
mml-miR-204-3p	GGCTGGGAAGGCAAAGGGACGT	GTCGTATCCAGTGCGTGTCGTGGAGTCGGCAATTGCACTGGATACGACACGTCCC	AGTTAGGCTGGGAAGGCAAA
pha-miR-769	TGAGACCTCTGGGTTCTGAGCT	GTCGTATCCAGTGCGTGTCGTGGAGTCGGCAATTGCACTGGATACGACAGCTCA	TCAGTTGAGACCTCTGGGTTC
mml-miR-148a-5p	AAAGTTCTGAGACACTCCGACT	GTCGTATCCAGTGCGTGTCGTGGAGTCGGCAATTGCACTGGATACGACAGTCGG	TGGCGAAAGTTCTGAGACACT
mml-miR-135a-1-3p	ATATAGGGATTGGAGCCGTGGC	GTCGTATCCAGTGCGTGTCGTGGAGTCGGCAATTGCACTGGATACGACGCCACGG	CGCTCGATATAGGGATTGGAG
mml-miR-150-5p	TCTCCCAACCCTTGTACCAGTG	GTCGTATCCAGTGCGTGTCGTGGAGTCGGCAATTGCACTGGATACGACCACTGG	TGCTGTCTCCCAACCCTTGT
mml-miR-199a-3p	ACAGTAGTCTGCACATTGGTTA	GTCGTATCCAGTGCGTGTCGTGGAGTCGGCAATTGCACTGGATACGACTAACCAA	TCTCGCACAGTAGTCTGCACA
pha-miR-145	GTCCAGTTTTCCCAGGAATCCCT	GTCGTATCCAGTGCGTGTCGTGGAGTCGGCAATTGCACTGGATACGACAGGGATT	ACGTGTCCAGTTTTCCCAGG
mml-miR-27a-5p	AGGGCTTAGCTGCTTGTGAGCA	GTCGTATCCAGTGCGTGTCGTGGAGTCGGCAATTGCACTGGATACGACTGCTCAC	GTGACAGGGCTTAGCTGCTT
MicroRNA U6		AACGCTTCACGAATTTGCGT	CTCGCTTCGGCAGCACA

**Table 2:** Distribution of sRNAs in PEDV-infected and uninfected samples.

Category	Infected	Uninfected
Raw reads	30,572,744/26,165,923/27,571,400	27,070,230/21,158,763/26,987,232
Clean reads	24,269,195/21,385,578/22,875,953	20,920,004/16,414,523/20,535,191
miRNAs’ reads	1,673,115/1,253,338/675,418(Total) 2074/2003/1752(unique)	1,047,301/788,983/1,212,878(Total) 1386/1208/1352(unique)
known miRNAs	441/415/396	352/326/346
novel miRNAs	310/306/290	210/181/208
rRNA reads	189,890/164,385/185,472	115,653/98,041/103,065
tRNA reads	29,156/30,890/14,333	13,765/6017/8148
snRNA reads	27,653/31,092/33,635	36,342/26,198/34,540
Cis-region reads	55,409/67,613/84,276	86,221/61,990/85,314
other_Rfam_RNA	73,478/74,761/99,704	76,065/61,225/70,461
unannotated	9,493,236/8,307,505/8,358,603	9,414,281/7,302,434/9,459,270

## Data Availability

The sequence data was deposited in the GenBank database (Accession number PRJNA826684).
